# Colorectal Cancers: An Update on Their Molecular Pathology

**DOI:** 10.3390/cancers10010026

**Published:** 2018-01-20

**Authors:** Kentaro Inamura

**Affiliations:** Division of Pathology, The Cancer Institute, Japanese Foundation for Cancer Research, 3-8-31 Ariake, Koto-ku, Tokyo 135-8550, Japan; kentaro.inamura@jfcr.or.jp; Tel.: +81-3-3570-0111 (ext. 5604); Fax: +81-3-3570-0558

**Keywords:** chromosomal instability, colorectal cancer, microsatellite instability, molecular characterization, personalized therapy

## Abstract

Colorectal cancers (CRCs) are the third leading cause of cancer-related mortality worldwide. Rather than being a single, uniform disease type, accumulating evidence suggests that CRCs comprise a group of molecularly heterogeneous diseases that are characterized by a range of genomic and epigenomic alterations. This heterogeneity slows the development of molecular-targeted therapy as a form of precision medicine. Recent data regarding comprehensive molecular characterizations and molecular pathological examinations of CRCs have increased our understanding of the genomic and epigenomic landscapes of CRCs, which has enabled CRCs to be reclassified into biologically and clinically meaningful subtypes. The increased knowledge of the molecular pathological epidemiology of CRCs has permitted their evolution from a vaguely understood, heterogeneous group of diseases with variable clinical courses to characteristic molecular subtypes, a development that will allow the implementation of personalized therapies and better management of patients with CRC. This review provides a perspective regarding recent developments in our knowledge of the molecular and epidemiological landscapes of CRCs, including results of comprehensive molecular characterizations obtained from high-throughput analyses and the latest developments regarding their molecular pathologies, immunological biomarkers, and associated gut microbiome. Advances in our understanding of potential personalized therapies for molecularly specific subtypes are also reviewed.

## 1. Introduction

Colorectal cancers (CRCs) represent a group of molecularly heterogeneous diseases that are characterized by a range of genomic and epigenomic alterations [1,2,3,4,5,6,7,8,9,10,11,12,13,14,15,16,17]. Our increasing understanding of the molecular pathological epidemiology of CRCs has enabled us to refine their classification from a heterogeneous group of diseases with variable clinical outcomes into characteristic molecular subtypes, a development that will allow personalized therapies to be implemented and improve the management of patients with CRC [18,19,20,21,22,23,24,25,26,27,28]. Knowledge regarding the molecular landscapes of CRCs is rapidly increasing; therefore, this review provides a perspective on recent updates regarding the molecular pathological epidemiology of CRCs. In addition, advances in our understanding of potential personalized therapies based on molecular-specific subtypes are discussed.

## 2. Classification by Molecular Subtype

Recent data regarding the comprehensive molecular characterizations of CRCs, including The Cancer Genome Atlas (TCGA) and consensus molecular subtype (CMS) classifications, have increased our understanding of the genomic and epigenomic landscapes of CRCs and have enabled their classification into various subtypes according to their distinct molecular pathologies and clinical features. In this section, recently identified CRC subtypes are reviewed on the basis of their comprehensive molecular characterizations.

### 2.1. Integrated Molecular Characterization (TCGA Classification)

In 2012, the TCGA research network conducted a comprehensive molecular characterization of 224 cases with CRC and analyzed exome sequences, DNA copy number, promoter methylation, and messenger RNA and microRNA expression patterns [1]. A subset of these samples, represented by 97 cases, was examined by whole-genome sequencing. Tumors with mutation rates of >12 mutations per 10^6^ bases (median number of total mutations, 728), which represented 16% of the total number of cases examined, were designated as hypermutated CRCs, whereas tumors with mutation rates of <8.24 mutations per 10^6^ bases (median number of total mutations, 58) were termed as non-hypermutated CRCs (84%). Among the hypermutated CRCs, 75% were enriched for microsatellite instability (MSI), *MLH1* methylation, and CpG island methylator phenotype (CIMP), whereas the remaining 25% presented with somatic mismatch repair (MMR) gene and polymerase ε (*POLE*) mutations, showing mutation rates of >40 mutations per 10^6^ bases.

The non-hypermutated CRCs were enriched for somatic mutations in the *APC* (81%), *TP53* (60%), *KRAS* (43%), *PIK3CA* (18%), *FBXW7* (11%), *SMAD4* (10%), *TCF7L2* (9%), *NRAS* (9%), *FAM123B* (7%), *CTNNB1* (*β-catenin*) (5%), *ACVR1B* (4%), and *SOX9* (4%) genes. *FAM123B* (also known as *WTX*) is an X-linked negative regulator of WNT signaling, and most of its mutations involve loss of function. In hypermutated CRCs, *ACVR2A* (63%), *APC* (51%), *TGFBR2* (51%), *BRAF* (46%), *MSH3* (40%), and *MSH6* (40%) genes were frequent mutation targets. Two genes that were frequently mutated in non-hypermutated CRCs were less frequently mutated in hypermutated CRCs: *TP53* (60% vs. 20%) and *APC* (81% vs. 51%).

All non-hypermutated CRCs were characterized as being microsatellite stable (MSS) and were more frequently associated with somatic copy number alterations (SCNAs), indicating that this group is enriched for chromosomal and sub-chromosomal changes.

The WNT signaling pathway was activated in 93% of non-hypermutated CRCs and 97% of hypermutated CRCs, an activation that involved either *APC* inactivation or *CTNNB1* activation, together with changes in many other genes involved in regulating this pathway, including *FBXW7*, *FAM123B*, *SOX9*, and *TCF7L2*. The TGF-β pathway was deregulated in 27% of non-hypermutated CRCs and 87% of hypermutated CRCs. Nearly all CRCs that were examined displayed dysregulation of MYC transcriptional targets because of the activation of MYC by activated WNT signaling and/or inactivation of the TGF-β pathway, indicating an important role for MYC in colorectal carcinogenesis.

This TCGA study suggested a number of therapeutic approaches to CRCs, including the use of WNT signaling and CTNNB1 inhibitors, which were shown to be promising in several studies [29,30]. Moreover, several proteins in the MAPK and PIK3 (PI3K) pathways, including IGF, IGFR, ERBB2, ERBB3, MEK, AKT, and MTOR, were considered to be potential targets for inhibition [1].

Proteomes of CRCs were analyzed using CRC cases that were characterized by TCGA in 2012. The integrated proteogenomic analyses that were performed [2] demonstrated a functional context for the observed genetic and epigenetic alterations, with relatively few extending to the protein level [2,4]. The genomic and proteomic approach revealed the importance of chromosome 20q amplification, including *HNF4A*, *TOMM34*, and *SRC*. Although HNF4A is a transcription factor that plays a key role in normal gastrointestinal development, there are contradictory studies regarding whether *HNF4A* acts as an oncogene or a tumor suppressor gene in CRC [31]. *TOMM34* is frequently overexpressed in CRCs and is involved in the growth of CRC cells [32], whereas *SRC* encodes a non-receptor tyrosine kinase that is implicated in colorectal carcinogenesis [33].

### 2.2. CRC Gene Expression Profiling (CMS Classification)

**Figure 1:**
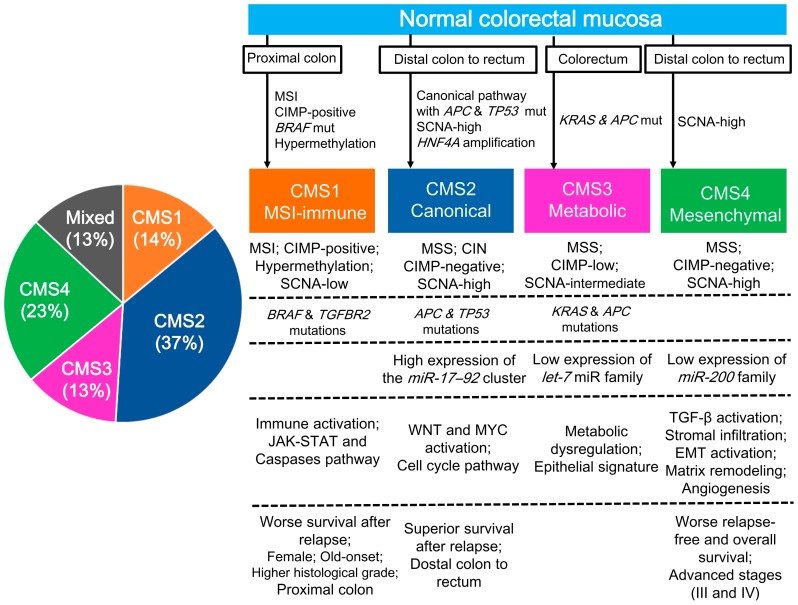
The taxonomy of colorectal cancer according to the Colorectal Cancer Subtyping Consortium, reflecting biological differences in the gene expression-based molecular subtypes [3]. CIMP, CpG island methylator phenotype; CIN, chromosomal instability; CMS, consensus molecular subtype; EMT, epithelial–mesenchymal transition; MSI, microsatellite instability; MSS, microsatellite stable; SCNA, somatic copy number alteration.

Although several studies conducted gene expression profiling for categorizing CRCs into subtypes, the results showed little agreement with each other and did not lead to a useful single classification [4,34,35,36]. Therefore, members of the Colorectal Cancer Subtyping Consortium decided to combine their genomic datasets comprising 4151 samples, including the TCGA source, to generate a consensus molecular subtyping by applying unsupervised clustering techniques [3]. From this, four CMSs were established (Figure 1). CMS1 (MSI immune, 14%) is characterized as showing MSI and immune activation; having a CIMP-positive, SCNA-low, *BRAF* mutant phenotype; and occurring in older and female patients in the proximal colon. CMS2 (canonical, 37%) is characterized as exhibiting MSS, chromosomal instability (CIN), and WNT/MYC pathway activation; having a CIMP-negative and SCNA-high phenotype; showing the presence of *APC* and *TP53* mutations; occurring in the distal colon to the rectum; and showing superior survival after a relapse. CMS3 (metabolic, 13%) is characterized as showing MSS, having a CIMP-low and SCNA-intermediate phenotype, showing the presence of *KRAS* and *APC* mutations, and exhibiting an epithelial signature and metabolic dysregulation. CMS4 (mesenchymal, 23%) is characterized as exhibiting MSS, being a CIMP-negative and SCNA-high phenotype, occurring at advanced stages, and showing poorer overall survival and signatures of TGF-β activation, stromal infiltration, epithelial–mesenchymal transition activation, matrix remodeling, and angiogenesis. Samples with mixed features (13%) were found to possibly represent either a transition phenotype or intratumoral heterogeneity. Although this CMS classification system was not a therapeutic stratifier, this subtyping by large datasets facilitated a better understanding of the broad biological groups comprising CRC.

### 2.3. CRC Subtypes Classified by Key Molecular Features

**Figure 2:**
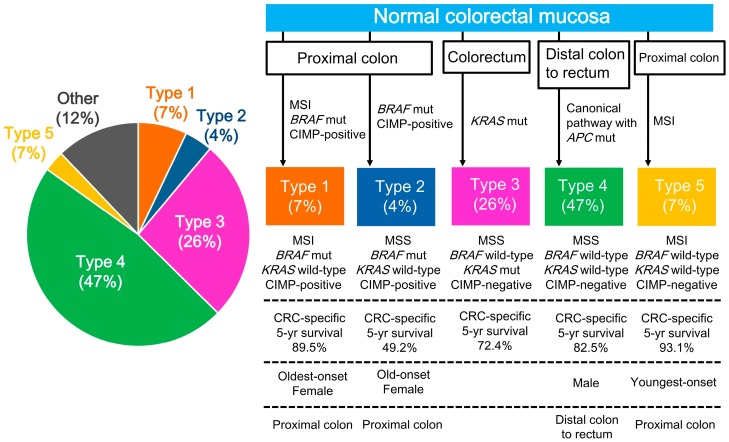
Categorization into five subtypes based on MSI and CIMP status and presence of *BRAF* and *KRAS* mutations [8]. CIMP, CpG island methylator phenotype; CIN, chromosomal instability; CMS, consensus molecular subtype; CRC, colorectal cancer; EMT, epithelial–mesenchymal transition; MSI, microsatellite instability; MSS, microsatellite stable; SCNA, somatic copy number alteration.

Although CRC is a biologically heterogeneous disease, categorization of colon cancers into distinct subtypes using a combination of key molecular features could provide insights regarding the varying clinical outcomes. Using a cohort of patients with stage III colon cancer in an adjuvant chemotherapy trial, Sinicrope et al. demonstrated that the combination of *KRAS* and *BRAF^V600E^* mutations with a DNA MMR status categorized colon cancers into five subtypes with distinct clinicopathological features, including clinical outcomes [7,10]. MMR-proficient tumors with *BRAF* or *KRAS* mutations, comprising 42% of all cases, exhibited higher mortality rates than tumors without this phenotype. *BRAF* wild-type, *KRAS* wild-type, and MMR-proficient tumors, comprising 49%, were the most prevalent subtype in the cohort and were associated with better survival than tumors that lacked this phenotype [7,10]. Using a population-based cohort of patients with stage I–IV CRCs, Phipps et al. demonstrated that the combination of statuses of MSI and CIMP and mutations of *BRAF* and *KRAS* enabled CRCs to be categorized into five subtypes with distinct clinicopathological features (Figure 2) [8]. Of the five subtypes, type 5 CRCs, comprising 7% of all cases and defined as showing MSI and having a *BRAF* wild-type, *KRAS* wild-type, and CIMP-negative phenotype, showed the lowest mortality rates and were clinicopathologically characterized based on their occurrence in the proximal colon and showed youngest onset. Type 4 CRCs (47%), defined as exhibiting MSS and having a *BRAF* wild-type, *KRAS* wild-type, CIMP-negative phenotype, represented the most prevalent subtype and were clinicopathologically characterized by their canonical pathway with *APC* mutations, and their occurrence in men and in the distal colon to rectum region. Type 4 mostly corresponds to CMS2 (canonical subtype) in the CMS classification [3]. Type 2 CRCs, comprising 4% of cases examined and defined as showing MSS and having a *BRAF* mutant, *KRAS* wild-type, CIMP-positive phenotype, showed the highest mortality rates and were clinicopathologically characterized by their occurrence in females and in the proximal colon and showed late age onset. These two studies suggest that categorization based on key molecular features of CRCs is useful for understanding the biological features of CRC and for predicting clinical outcomes. Validating the study by Phipps et al. [8], a recent study reported that CIMP positivity could be used to stratify patients with poor prognosis having MSS and *BRAF* mutant CRCs, which correspond to type 2 CRCs in the study by Phipps et al. [37].

## 3. Molecular Biomarkers

Key molecular biomarkers are important for understanding the biological heterogeneity of CRCs and for classifying CRCs into subtypes that can be used to predict prognosis, treatment response, and recurrence risk. These key molecular features or pathways can potentially represent targets for personalized therapies.

### 3.1. CIN

The acquisition of genomic instability is a distinct feature of tumorigenesis, and there are three distinct pathways in colorectal carcinogenesis: CIN, MSI, and CIMP [38]. CIN is the most common feature of CRCs (75‒85%) compared with MSI or CIMP [1]. Although substantial progress has been made in identifying the causes of CIN in CRCs, its underlying mechanisms remain unknown [38,39,40]. Possible mechanisms include alterations in chromosome segregation, telomere dysfunction, and DNA damage response, which affects critical genes such as *TP53* and *APC*. The loss-of-function mutations of *TP53*, which is the main cell cycle checkpoint gene, cause uncontrolled entry in the cell cycle [38]. CRCs with CIN are characterized by the presence of extensive SCNAs throughout the genome and result in aneuploidic tumors and loss of heterozygosity [1,9]. *APC* mutations, which are associated with defects in chromosomal segregation [41], are also strongly associated with CIN [38], and thus are likely to lead to CIN and promote cancer progression in CRCs. *APC* forms part of the WNT signaling pathway [7], and its inactivation results in an increase in nuclear CTNNB1 expression and cell proliferation. Thus, the WNT signaling pathway plays a gatekeeper role in CIN CRCs.

### 3.2. MSI

MSI, defined by the National Cancer Institute (NCI) panel markers, BAT26, BAT25, D5S346, D2S123, and D17S250, is a biomarker for defective DNA MMR function in CRCs. According to the classification based on these markers, MSI tumors exhibit instability in two or more markers, whereas MSS tumors show instability in no more than one marker [42]. When CRCs with instability in <30% of markers (MSI-low) were compared with MSS CRCs, MSI-low CRCs did not show any prognostic values compared with MSS CRCs. Therefore, MSI-low CRCs were classified in the same subtype as MSS CRCs [43]. MSI is observed in approximately 15% of sporadic CRCs, most consistently with the frequency of hypermutated CRCs, to which they categorically belong. In addition, frameshift mutations have been detected in the NCI consensus panel of biomarkers; however, multiple other mutations, including point mutations, also occur in the MMR-defective status. The most prevalent cause of the MMR-defective status in sporadic CRCs is the aberrant biallelic hypermethylation of the DNA MMR gene *MLH1*, which prevents its gene expression. MSI generally results from the inactivation of the MMR genes through aberrant promoter hypermethylation (80% of MSI CRCs; most frequently *MLH1*) or mutations in the MMR genes, comprising *MLH1*, *MSH2*, *MSH6*, and *PMS2* (20% of MSI CRCs). Although CRCs can be analyzed by polymerase chain reaction to detect the presence of MSI, immunohistochemistry can be used to easily evaluate MSI status by demonstrating the absence of a DNA MMR protein. MSI CRCs are mostly enriched for the epigenetic inactivation of the *MLH1* gene, have a CIMP-positive and SCNA-low phenotype, show high frequency of the *BRAF^V600E^* mutation and a low frequency of *APC* and *TP53* mutations, and are characterized by their occurrence in females at a late age and in the proximal colon, with poor tumor differentiation and mucinous/signet-ring cell histology. A recent comprehensive molecular characterization revealed that *RNF43* is frequently mutated in CRCs and endometrial cancers [6]. *RNF43* encodes an E3 ubiquitin ligase that negatively regulates WNT signaling. Truncating mutations of *RNF43* are enriched in MSI CRCs and mutually exclusively occur with inactivating *APC* mutations in CRCs. Moreover, an additional study demonstrated the significant co-occurrence of *RNF43* and *BRAF* mutations in the serrated neoplasia pathway [44].

Because nearly all hypermutated CRCs demonstrate a deregulated WNT signaling pathway [1], this pathway is believed to play a gatekeeper role even in MSI CRCs similar to CIN CRCs. MSI CRCs are clinically characterized as having a favorable prognosis. Furthermore, MSI is a possible marker of sensitivity to therapy with 5-fluorouracil (5-FU). The responsiveness to 5-FU in MSI CRCs seems to depend on the stages of CRCs. Stage II MSI CRCs lack the sensitivity to 5-FU-based adjuvant chemotherapy. In stage III MSI CRCs, the sensitivity to 5-FU-based adjuvant chemotherapy or the standard adjuvant chemotherapy remains controversial, and further studies are required [45,46,47,48]. Recent studies suggest that MSI is a marker of good response to 5-FU treatment, particularly in the presence of large deletions in *HSPH1* (*HSP110*) [49,50].

In May 2017, the US Food and Drug Administration (FDA) granted the accelerated approval to pembrolizumab, a monoclonal anti-PD-1 (PDCD1) antibody, for patients with MSI or MMR-deficient solid tumors. This is the first time that FDA has approved a cancer treatment based on a common biomarker rather than an organ-based approach [51]. MSI causes increased somatic mutations in tumor cells, leading to molecular and biological changes, including high tumor mutational burden, increased expression of neoantigens, and abundant tumor-infiltrating lymphocytes. These changes are associated with an increased sensitivity to checkpoint inhibitor drugs [23,52,53,54].

### 3.3. CIMP

Epigenetic instability, which is responsible for CIMP, is another prevailing feature of CRCs. The important feature of CIMP-positive tumors is the hypermethylation of promoters of cancer-related genes, which leads to genetic silencing and an absence of protein expression. In CRCs, genetic and epigenetic events are not exclusive, and both cooperate in CRC development, although methylation events are more frequently observed than point mutations. Definitions of CIMP varies substantially among studies with respect to examined foci of methylation and cut-off values for CIMP-positive and CIMP-negative [55]. However, no specific CIMP definitions have been confirmed yet to be superior to the others. A recent study demonstrated that the CIMP status did not show any relationship with CRC prognosis. However, combinations of CIMP with MSI or *BRAF* mutation were associated with CRC survival, although these associations were observed regardless of CIMP status [56]. Because most CIMP-positive CRCs exhibit an MSI phenotype, clinicopathological features of CIMP-positive CRCs overlap with MSI CRCs. Similar to CIMP-positive MSI CRCs, CIMP-positive MSS CRCs are characterized by a high frequency of *BRAF^V600E^* mutation; occurrence at a later age, in females, and in the proximal colon, and with poor tumor differentiation. *APC* mutations and activation of the WNT/CTNNB1 signaling pathway are inversely associated with CIMP. 

CIMP-positive CRCs arise from a serrated precursor lesion, such as sessile serrated polyp/adenoma [57]. In line with the difficulty of endoscopically detecting a precursor of CIMP-positive CRCs such as sessile serrated polyp/adenoma, CRC diagnosed within five years after colonoscopy is likely to have a CIMP-positive phenotype [58].

### 3.4. POLE Mutations

*POLE* mutations were identified in ultramutated CRCs in the TCGA study [1]. Seven of 30 (23%) hypermutated CRCs lacked MSI, CIMP, and *MLH1* hypermethylation but had somatic mutations in *POLE* and missense or nonsense (but not frameshift) mutations in one or more DNA MMR genes and were designated as ultramutated CRCs [1,59]. *POLE* encodes one of three polymerases—POLA1, POLD1, and POLE—that are responsible for replicating nuclear DNA and that are involved in the synthesis stage of the DNA repair process; they also play a key role in recombination [60,61]. Somatic *POLE* mutations apparently cause MSS ultramutated CRCs, unless two DNA MMR alleles of the same gene became mutated by chance. The importance of *POLE* mutations in tumorigenesis has been demonstrated in endometrial cancers and CRCs [61]. Germline mutations of *POLE*, likely to be the cause of predisposition to colorectal and other cancers, and those of *POLD1* map to equivalent sites in the proofreading (exonuclease) domain of *POLE* and *POLD1* and are predicted to cause a defect in the correction of mispaired bases inserted during DNA replication. As expected, tumors from carriers of *POLE* and *POLD1* germline mutations are MSS but tend to acquire base substitution mutations [62]. Tumors with *POLE* or *POLD1* mutations are characterized by an extremely high mutation frequency (>1 million per genome) despite MSS. Clinically, only a weak association exists between the presence of mutations in the exonuclease domain of *POLE*/*PODL1* and increased mortality in MSS CRCs [63]. In contrast to *POLE* and *POLD1*, *POLA1* mutations are rare and functionally impaired because of stringent selection [60].

Accumulating evidence indicates that MSI and MMR deficiency with high tumor mutational load can predict a response to the anti-PDCD1 antibody in metastatic CRC (mCRC) [20,23,52,53,54,64]. *POLE*-mutant CRC represents an ultramutated but MSS phenotype that is uniquely different from usual CRC with an MSS phenotype. Possibly because of the ultramutated phenotype with a high mutational load and increased expression of neoantigens, patients with treatment refractory mCRC that is characterized by an MSS phenotype and *POLE* mutations may show clinical responses to pembrolizumab [65].

### 3.5. LINE-1 Hypomethylation

Although DNA hypermethylation can inactivate tumor suppressor genes, global hypomethylation, an overall genome-wide reduction in DNA methylation content, also exerts an influence on colorectal carcinogenesis by inducing chromosomal instability, leading to elevated mutation rates [66]. Genomic DNA hypomethylation is likely to be accompanied by repetitive transposable DNA elements such as the long interspersed nucleotide element-1 (LINE-1) or short interspersed nucleotide element. LINE-1 constitutes a substantial portion (approximately 18%) of the human genome [67], and the methylation level in LINE-1 correlates with global DNA hypomethylation status [68]. Activated LINE-1 retrotransposons lead to chromosomal instability, transcription of adjacent genes, gene disruption, and generation of gene transcripts involved in the regulation of gene expression or telomere maintenance [69,70].

LINE-1 hypomethylation in CRC is characterized by early age onset, family history of CRC, reduced mucinous/signet-ring cell component, showing MSS and CIN, and the presence of a CIMP-negative, *BRAF* wild-type phenotype [71]. Although LINE-1 hypomethylation is associated with a higher mortality, this association is stronger in MSI CRCs than in MSS CRCs. Tumor LINE-1 methylation level may be a useful prognostic biomarker for identifying aggressive carcinomas among MSI CRCs, which are usually associated with a favorable prognosis [72]. A somatic LINE-1 insertion in the *APC* gene, together with a point mutation in the second *APC* allele, was recently shown to initiate colorectal tumorigenesis through the classic two-hit CRC pathway [73]. The LINE-1 hypomethylation status of circulating cell-free DNA in plasma could be used as a potential biomarker for CRC, particularly for the early stage form [74].

### 3.6. RAS, BRAF, and PIK3CA Mutations in the MAPK/PIK3 Pathway

The MAPK and PIK3 (PI3K) pathways are both involved in cell proliferation. Alterations that affect these pathways contribute to providing proliferative advantages for tumor cells. Mutations of *KRAS*, *BRAF*, and *PIK3CA* are the most common to affect the MAPK/PIK3 pathways in colorectal tumorigenesis. Approximately 40% of CRCs harbor *KRAS* mutations [11,75,76]. In contrast, *NRAS* mutations were observed in just 2.5‒4.5% of CRCs [11,77]. *KRAS* and *NRAS* mutations predict resistance to anti-EGFR antibody therapy [78]. In addition, recent studies suggest that *BRAF* and *PIK3CA* mutations also contribute to the resistance to anti-EGFR antibody therapy [78,79,80].

The prognostic association of *KRAS* mutations in patients with CRC is conflicting [11,81,82]. *KRAS^G12C^* and *KRAS^G12V^* mutations may be independently associated with worse overall survival after diagnosis [76]. One study suggested that the adverse effect of *KRAS* mutations on survival is stronger in distal colon cancers than in proximal colon cancers [83]. In line with this study, *KRAS* or *BRAF* mutations may be associated with shorter overall survival in patients with MSS but not in those with MSI tumors [84]. In contrast, another study demonstrated that the survival of patients with stage II/III CRC might be predicted by CIN and MSI but not by specific driver mutations, including *KRAS*, *NRAS*, *BRAF*, and *PIK3CA* [81]. A recent nested case-control study suggested that *KRAS* mutant CRC risk, but not *KRAS* wild-type CRC risk, may be associated with low plasma adiponectin levels [85].

Approximately 8% of CRCs harbor a point mutation of *BRAF* that is mutually exclusive with *KRAS* mutations. *RAS* mutations are more present in *BRAF^D594G^* mutant CRCs than in *BRAF^V600E^* mutant CRCs. The *BRAF^V600E^* mutation, but not the *BRAF^D594G^* mutation, is associated with poor prognosis. More *BRAF^V600E^* mutant CRCs were found in the proximal colon compared with *BRAF^D594G^* mutant CRCs [86]. As with *RAS* mutations, *BRAF* mutant CRCs are less susceptible to anti-EGFR antibody therapy. In contrast to the pronounced response to *BRAF^V600E^* mutant melanoma, vemurafenib, a BRAF inhibitor, does not show a meaningful clinical activity in patients with *BRAF^V600E^* mutant CRC [87]. *BRAF* mutant CRCs are resistant to vemurafenib owing to EGFR-mediated re-activation of MAPK signaling [88,89]. In view of this evidence, the combination of BRAF inhibitor and MAP2K (MEK) inhibitor was applied to *BRAF* mutant CRCs, with modest activity being observed in a subset of patients with metastatic *BRAF^V600E^* mutant CRC [90,91].

*PIK3CA* mutations are present in 10‒20% of CRCs and are associated with other molecular alterations, including the *KRAS* mutant and CIMP-positive phenotype [11]. PIK3CA is an indispensable element of the PIK3 signaling pathway downstream of EGFR. The *PIK3CA* mutation activates the PIK3 signaling pathway, enhancing cell proliferation and eventually leading to carcinogenesis. As with *RAS* and *BRAF* mutations, *PIK3CA* mutations predict resistance to anti-EGFR antibody therapy. CRCs with *PIK3CA* mutations overexpress PTGS2 (COX2), which plays a critical role in regulating inflammatory responses by generating prostaglandins. Aspirin inhibits PTGS2 expression and downregulates the PIK3 signaling pathway. Regular use of aspirin appears to reduce the risk for PTGS2-overexpressing CRCs but not the risk for PTGS2 weakly expressing or PTGS2-absent CRCs [92]. Furthermore, regular aspirin use after CRC diagnosis is associated with a lower risk for mortality, particularly among individuals with PTGS2-overexpressing CRC [93]. Similar to PTGS2 expression, regular aspirin use was associated with lower mortality in patients with *PIK3CA* mutant CRC but not in those with *PIK3CA* wild-type CRC [21]. A recent population-based cohort study demonstrated that the association of aspirin use with improved survival differed according to PTGS2 expression but not according to *PIK3CA* mutation status [94]. Another recent study suggests that the regular use of nonsteroidal anti-inflammatory drugs (NSAIDs) is associated with improved survival in patients with *KRAS* wild-type CRC but not in those with *KRAS* mutant CRC [95]. Genome-wide single-nucleotide polymorphism (SNP) data suggested that the association of aspirin and/or NSAIDs with a lower risk for CRCs differs according to the genetic variation at two SNPs on chromosomes 12 and 15 [96]. The 15-hydroxyprostaglandin dehydrogenase (HPGD) is downregulated in CRCs and functions as a metabolic antagonist of PTGS2. *HPGD* mRNA expression levels in normal mucosa may serve as a biomarker that predicts a stronger benefit from aspirin chemoprevention [97].

### 3.7. WNT/APC/CTNNB1/TGF-β Pathway

Most sporadic CRCs show abnormal activation of the WNT pathway. Genetic disruption of *APC*, which leads to the activation of the WNT pathway, is a critical early genetic event in colorectal tumorigenesis. In the TCGA study, the WNT pathway was activated in >90% of both non-hypermutated and hypermutated CRCs [1]. Approximately 80% CRCs had *APC* mutations, whereas 5%‒10% CRCs exhibited mutations or epigenetic alterations in other WNT signaling components (e.g., *CTNNB1*) that similarly result in the activation of the WNT pathway [1,39]. APC is not only a critical negative regulator of the WNT pathway but also regulates chromosomal segregation, cellular differentiation, adhesion, migration, and apoptosis.

As a negative regulator of the WNT pathway, APC promotes the proteasomal degradation of CTNNB1, which is an important activator of the WNT pathway. If APC is inactivated by mutation, excess cytoplasmic CTNNB1 accumulates and translocates to the nucleus where CTNNB1 modulates a transcriptional shift, promoting the activation of *MYC* and many other oncogenes. The disruption of the WNT pathway dysregulates cell proliferation and normal differentiation of colonic epithelia, with adenomas progressing from low grade to high grade owing to the inactivation of other tumor suppressor genes. The transition from adenoma to invasive carcinoma is usually associated with the inactivation of the *TP53* tumor suppressor gene [38,39,40].

The TGF-β pathway plays a critical role in fundamental cellular processes, including cell growth, differentiation, and apoptosis. Chromosomal changes that involve TGF-β strongly contribute to the CIN pathway in colorectal tumorigenesis. The loss of chromosomal 18q is one of the main genomic alterations associated with the inactivation of the TGF-β pathway. Chromosome 18q encodes for two important tumor suppressor genes, *SMAD2* and *SMAD4*, the loss of which inactivates the TGF-β signaling pathway and promotes the evasion of apoptosis and cell proliferation. Nearly all CRCs display MYC activation by the inactivation of the TGF-β pathway and/or activated WNT signaling, indicating an important role for *MYC* in CRC [1]. In the normal colorectal or early CRC tissues, the TGF-β pathway serves as a tumor suppressor by inhibiting cell proliferation and immortalization, and inducing apoptosis; therefore, the inactivation of the TGF-β pathway promotes colorectal tumorigenesis. However, as tumors develop and progress, the tumor-suppressive effects of the TGF-β pathway are often lost. During the late stages of colorectal carcinogenesis, the TGF-β pathway switches to be oncogenic and its activation promotes cancer progression, invasion, and tumor metastasis [98,99,100].

### 3.8. TP53 Mutations

*TP53* is one of the most important tumor suppressor genes and is the main cell cycle checkpoint regulator [38]. *TP53* inactivation drives tumor progression, allowing excessive cell proliferation. Indeed, the transition from adenoma to invasive carcinoma is usually associated with *TP53* inactivation [38,39,40]. Loss of 17q, where *TP53* is located, is a frequent event in CRCs because it plays a critical role in the canonical adenoma–adenocarcinoma sequence. *TP53* is more frequently mutated in non-hypermutated CRCs than in hypermutated CRC, similar to the *APC* gene [1]. Of note, not only losses of TP53 activity but also “gain-of-function” *TP53*-mutants mediate tumor metabolic reprogramming, which promotes tumor progression and invasion [101].

### 3.9. Immune Biomarkers and the Microbiome

Immunotherapy has developed as a promising strategy for the treatment of various malignancies, including CRCs [19,102,103,104,105,106]. Emerging evidence indicates that immune checkpoint mechanisms play a critical role in suppressing the anti-tumor T-cell-mediated immune response in the tumor microenvironment. CD274 (PD-L1) is an immune modulator that promotes immunosuppression by binding to PDCD1 (PD-1) of T cells. CD274 engages in the negative regulation of the immune response through the PDCD1 receptor, and evading the host immune surveillance is an important strategy in cancer. Therapeutic antibodies that target PDCD1 and CD274 are effective in numerous malignancies, including CRCs [20,23]. Tumor CD274 expression is a potential biomarker of a better response to anti-PDCD1/CD274 therapies [107,108].

Recent studies have suggested the importance of complex associations between tumor molecular characteristics and immune cells in the tumor microenvironment [54,109]. Emerging evidence has suggested the interactive influences of tumor molecular features with the immune response to the tumor [110,111]. A recent study demonstrated an association of pro-inflammatory diets such as red and processed meats, with a higher risk for CRC subtypes with absent/low-lymphocytic reaction than CRC subtypes with high-lymphocytic reaction in the tumor microenvironment. The pro-inflammatory diet-associated CRC subtype was enriched in MSS, CIMP-low/negative, and *BRAF* wild-type phenotype [112]. The expression level of CD274 in tumors is inversely associated with the density of FOXP3-positive regulatory T cells, revealing the potential interactions between the immune checkpoint pathway and the host immunity in colorectal carcinogenesis [113]. For a survival association, the association of post-diagnosis aspirin use with better CRC-specific survival seems to be stronger in patients with CD274-low tumors than in those with CD274-high tumors [24]. Moreover, whereas tumors with a high neoantigen load and increased immunogenicity are likely to be a target of immunotherapy [114,115], tumors with a high neoantigen load were found to be associated with an increased lymphatic infiltration. In addition, immune cell-infiltrated CRCs were enriched in *HLA* mutations [110].

The microbiota is associated with tumor initiation and progression in CRC by affecting intestinal inflammation and modulating the tumor-related signaling pathway [116,117,118,119]. In CRC tissues, a greater abundance of *Fusobacterium nucleatum* is detected compared to matched non-malignant colorectal tissues [120,121]. Indeed, the tumorigenic activity of CRC cells increased after infection with *Fusobacterium nucleatum* via the activation of TLR4 signaling to MYD88 and then leading to NFKB activation and increased *MIR21* (*miR21*) expression [122]. With regard to a therapeutic association, *Fusobacterium nucleatum* orchestrates a molecular network of TLRs, microRNAs, and autophagy to promote the chemoresistance of CRCs [123].

CRC microbiomes are associated with tumor CMSs [3,124]. CMS1 (MSI-immune), which is characterized as having the MSI, CIMP-positive, SCNA-low, *BRAF* mutant, and immune activation phenotype, was enriched for *F. nucleatum*. Meanwhile, CMS2 (canonical), characterized as having the MSS, CIMP-negative, CIN, SCNA-high phenotype, with mutations in *APC* and *TP53*, and the WNT/MYC pathway activation, was enriched for *Selenomas* and *Prevotella* species [124].

## 4. Conclusions and Future Directions

This review shows our current knowledge of the molecular pathologies of CRCs, including updated comprehensive molecular characterizations, advances in our understanding of molecular pathologies, identification of immune biomarkers for potential targeted immunotherapies, and influence of the gut microbiome on the tumor microenvironment. Recent advances in our understanding of the molecular characteristics of CRCs will potentially permit their evolution from a poorly understood, heterogeneous group of diseases with variable clinical courses and therapeutic responses toward more specific, molecularly characterized subtypes. Moreover, the identification of key molecular features or pathways that are specific to a certain CRC subtype may represent potential therapeutic targets, enabling the implementation of tailored therapies and better patient management. Although molecular characteristics and classifications of CRC have been identified in detail as potential targets of tailored therapies or prognostic predictors, the increased knowledge is currently less useful with respect to targeted therapy and prognostication in the clinical practice [125]. The identified associations between the molecular classifications and clinical factors often lack the required validation. Molecular features are not yet adopted as targets of personalized therapy nor integrated into the TNM system. We really need to press forward the clinical translation and precision medicine to reduce the number of unsuccessful treatments of CRC patients and CRC-related deaths.

## Abbreviations

The following abbreviations are used in this manuscript:

5-FU	5-fluorouracil
CIMP	CpG island methylator phenotype
CIN	chromosomal instability
CMS	consensus molecular subtype
CRC	colorectal cancer
EMT	epithelial–mesenchymal transition
FDA	Food and Drug Administration
LINE-1	long interspersed nucleotide element-1
mCRC	metastatic colorectal cancer
MMR	mismatch repair
MSI	microsatellite instability
MSS	microsatellite stable
NCI	National Cancer Institute
NSAID	nonsteroidal anti-inflammatory drug
SCNA	somatic copy number alteration
SNP	single-nucleotide polymorphism
TCGA	The Cancer Genome Atlas
